# Co(III) and Ni(II) Complexes Containing Bioactive Ligands: Synthesis, DNA Binding, and Photocleavage Studies

**DOI:** 10.1155/2007/36497

**Published:** 2007-02-11

**Authors:** M. C. Prabhakara, B. Basavaraju, H. S. Bhojya Naik

**Affiliations:** ^1^Department of PG Studies and Research in Industrial Chemistry, School of Chemical Sciences, Kuvempu University, Shankaraghatta, Shimoga, Karnataka 577 451, India; ^2^Department of Biotechnology, GM Institute of Technology, Davanagere, Karnataka 577 006, India

## Abstract

DNA binding and photocleavage characteristics of a series of mixed ligand complexes of the type [M(bpy)_2_qbdp](PF_6_)_n_·xH_2_O (where M = Co(III) or Ni(II), bpy = 2.2′-bipryidine, qbdp = Quinolino[3,2-b]benzodiazepine, n = 3 or 2 and x = 5 or 2) have been investigated. The DNA binding property of the complexes with calf thymus DNA has been investigated by using absorption spectra, viscosity measurements, as well as thermal denaturation studies. Intrinsic binding constant (*K*_b_) has been estimated under similar set of experimental conditions. Absorption spectral studies indicate that the Co(III) and Ni(II) complexes intercalate between the base pairs of the CT-DNA tightly with intrinsic DNA binding constant of 1.3 × 10^6^ and 3.1 × 10^5^ M^−1^ in Tris-HCl buffer containing 50 mM NaCl, respectively. The proposed DNA binding mode supports the large enhancement in the relative viscosity of DNA on binding to quinolo[3,2-b]benzodiazepine. The oxidative as well as photo-induced cleavage reactions were monitered by gel electrophoresis for both complexes. The photocleavage experiments showed that the cobalt(III) complex can cleave pUC19 DNA effectively in the absence of external additives as an effective inorganic nuclease.

## 1. INTRODUCTION

The interaction and reaction of metal complexes with DNA have long been the subject of intense investigation in relation to the development of new reagents for biotechnology and medicine. Studies of small molecules, which react at specific sites along a DNA strand as reactive models for protein-nucleic acid interactions, provide routes towards rational drug design as well as means to develop sensitive chemical probes for DNA. A number of metal complexes have been used as probes of DNA structure in solution, as agents for mediation of strand scission of duplex DNA and as chemotherapeutic agents [1–7]. In this regard, mixed-ligand metal complexes have been found to be particularly useful because of their potential to bind DNA *via* a multitude of interactions and to cleave the duplex by virtue of their intrinsic chemical, electrochemical, and photochemical reactivities [8–15]. Prominent among the various mixed-ligand metal complexes employed so far in studies with DNA are those metallointercalators which incorporate either 2.2′-bipyridine(bpy)/1.10 phenanthroline or a modified bipyridine/phenanthroline moiety or aromatic heterocyclic ring as a ligand. A singular advantage in using these metallointercalators for such studies is that the ligands or the metal ion in them can be varied in an easily controlled manner to facilitate individual applications [16–18]. Although DNA interactions of number of mixed ligand complexes have previously appeared in the literature, still there is scope to design and study small molecules containing mixed ligand with the same or different metal ions as new chemical nucleases.

In continuation of our work on studies of nuclease activity of mixed-ligand complexes, we wish to explore the binding and oxidative as well as photocleavage activities of mixed ligand complexes of Co(III) and Ni(II) containing bipyridine and condensed quinoline derivative ligand.

## 2. EXPERIMENTAL

All reagents and solvents were of AR grade, purchased commercially. All the solvents were purified and used. *o*-Phenylenediammine, CoCl_2_ · 6H_2_O, NiCl_2_ · 6H_2_O and 2.2′-bipyridine, ammonium hexaflurophosphate (NH_4_PF_6_), and Tris-HCl buffer were purchased from Qualigens (Mumbai, India). Calf thymus DNA (CT-DNA) and pUC19 DNA were purchased from Bangalore Gene, Bangalore, India. Tris-HCl buffer (5 mM Tris-HCl, 50 mM NaCl, pH-7.2, Tris = Tris(hydroxymethyl) amino methane) solution was prepared using deionised double distilled water.

### 2.1. Synthesis of quinolino[3,2-b]benzodiazepine(qbdp)

**Figure 1:**
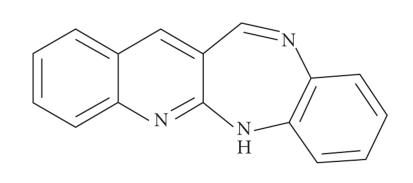
Quinolino[3,2-b]benzodiazepine(qbdp).

2-Chloro-3-quinolinecarbaldehyde (0.958 g, 5 mmo1) dissolved in small amount of acetic acid was taken in a 100 mL borosil beaker. *o*-Phenylenediammine (0.541 g, 5 mmol) and a pinch of potassium iodide were then added. The whole mixture was made into slurry and was irradiated by placing the beaker in a microwave oven for about 10 minutes. The completion of the reaction was monitored by TLC. The product obtained was poured into ice-cold water, the solid separated was filtered, dried, recrystallized, and its physical constants were measured (see Figure 1).

*Analysis*: calculated for C_16_H_11_N_3_; C, 78.35; H, 4.52; N, 17.13%; found: C, 78.52; H, 4.76; N, 17.35%; IR (KBr, cm^−1^): 3330 (N−H); 1576 (C=C); 1658 (C=N); 2924 (C−H, aromatic); ^1^H NMR (DMSO-d_6_): *δ* 10.65 (s, 1H, NH); 8.4 (s, 1H, H−C=N); 7.2–7.8 (m, 9H, Ar-H); MS: *m/z* 248.5.

### 2.2. Synthesis of metal complexes

#### 2.2.1. Synthesis of Co(III) and Ni(II) complexes

The complexes [Co(bpy)_2_Cl_2_]Cl · 3H_2_O and [Ni(bpy)_2_Cl_2_] were prepared as reported previously [19, 20].

#### 2.2.2. Synthesis of [Co(bpy)_2_(qbdp)](PF_6_)_3_·5H_2_O (1)

**Figure 2:**
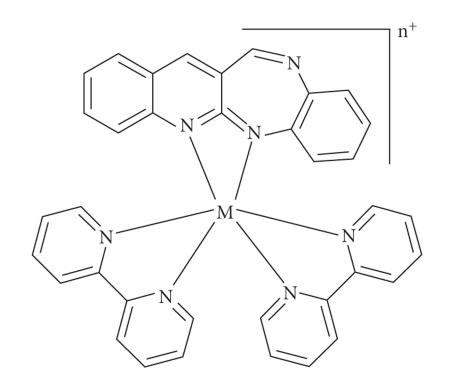
Probable structure of [M(bpy)_2_qbdp] complex, where M = Co(III) or Ni(II), n = 2 or 3.

[Co(bpy)_2_Cl_2_]Cl · 3H_2_O (0.53 g, 1 mmol) and qbdp (0.245 g, 1 mmol) were taken in a solvent mixture containing 20 mL ethylene glycol and 5 mL methanol. The resulting mixture was refluxed for 4 hours, allowed to cool, and then filtered. The desired complex was precipitated upon addition of a methanolic solution of NH_4_PF_6_ to the filtrate. The complex was filtered, further and dried under vacuum and recrystallized from an acetone-diethyl ether mixture (see Figure 2). *Analysis*: yield of complex 0.99 g (74%); calculated for C_36_H_36_N_7_O_5_P_3_F_18_Co: C, 71.42; H, 5.24; N, 14.58; Co, 8.76: Found: C, 70.98; H, 5.86; N, 13.84; Co, 9.89. IR, KBr pellets (cm^−1^): 839, 1321, 1431, 1581, 1600. ^1^H NMR, *δ*ppm (DMSO·d_6_, 200 MHz), ppm (TMS): 9.92 (d, 2H), 9.18 (m, 4H), 8.92 (d, 2H), 8.60 (d, 6H m), 8.29 (d, 2H), 8.00 (m, 8H), 7.70 (d, 4H); MS: *m/z* 658.

#### 2.2.3. Synthesis of [Ni(bpy)_2_(qbdp)](PF_6_)_2_·2H_2_O (2)

A methanolic solution of [Ni(bpy)_2_Cl_2_] (0.44 g, 1 mmol) was added to a mixture of methanolic solution of qbdp (0.245 g, 1 mmol) and ethylene glycol (25 mL). The resulting mixture was refluxed for 4 hours, allowed to cool, and then filtered. The crude complex was precipitated upon addition of saturated methanolic solution of NH_4_PF_6_ to the filtrate. The complex was filtered, further and dried under vacuum and recrystallized from an acetone-diethyl ether mixture (see Figure 2). *Analysis*: yield of complex 0.81 g (80%); calculated for C_36_H_30_N_7_O_2_P_2_F_12_Ni: C, 72.14; H, 4.85; N, 12.96; Ni, 10.56: Found: C, 71.44; H, 5.25; N, 14.58; Ni, 9.73. IR, KBr pellets (cm^−1^): 839, 1333, 1420, 1587, 1605; MS: *m/z* 657. *μ*_eff_ = 2.76 ± 0.02 B.M.

### 2.3. Spectral measurements

Melting points were determined in open capillaries and are uncorrected. Microanalyses (C, H, and N) were performed in Carlo-Erba 1106-model 240 Perkin-Elmer analyzer. The molar conductivities in DMF (10^−3^ M) at room temperature were measured using an Equiptronics digital conductivity meter. Magnetic measurements were carried out by the Gouy method at room temperature (28 ± 2°C) using Hg[Co(SCN)_4_] as calibrant. IR spectra were recorded with Shimadzu model FT-IR spectrophotometer by using KBr pellets. ^1^H-NMR spectra were recorded on a Bruker FT-NMR spectrometer (300 MHz) at 25°C in DMSO with TMS as the internal reference. FAB-MS spectra were recorded with a JEOL SX 102/DA-6000 mass spectrometer/data system. UV visible absorption spectra were recorded using Shimadzu model UV spectrophotometer at room temperature. Viscosity measurements were carried out on semimicro dilution capillary viscometer (Viscomatic Fica MgW) with a thermostated bath D40S at room temperature. Thermal denaturation studies were carried out with a Perkin-Elmer Lambda 35 spectrophotometer.

### 2.4. DNA binding and cleavage experiments

The concentration of CT DNA per nucleotide [C(p)] was measured by using its known extinction coefficient at 260 nm (6600 M^−1^cm^−1^) [21]. The absorbance at 260 nm (A_260_) and at 280 nm (A_280_) for CT DNA was measured to check its purity. The ratio A_260_/A_280_ was found to be 1.84, indicating that CT DNA was satisfactorily free from protein. Buffer [5 mM tris(hydroxymethyl)aminomethane, tris, pH 7.2, 50 mM NaCl] was used for the absorption, viscosity, and thermal denaturation experiments.

Absorption titration experiments were carried out by varying the DNA concentration (0–100 *μ*M) and maintaining the metal-complex concentration constant (30 *μ*M). Absorption spectra were recorded after each successive addition of DNA and equilibration (approximately 10 minutes). For both the complexes **(1)** and **(2)**, the observed data were then fit in to (1) to obtain the intrinsic binding constant, *K*_b_ [22]:

(1) $$\frac{\left[\text{DNA}\right]}{\left(\varepsilon_{a}-\varepsilon_{f}\right)}=\frac{\left[\text{DNA}\right]}{\left(\varepsilon_{b}-\varepsilon_{f}\right)}+\frac{1}{K_{b}\left(\varepsilon_{b}-\varepsilon_{f}\right)}$$,

where *ɛ*_a_, *ɛ*_f_, and *ɛ*_b_ are the apparent, free, and bound metal-complex extinction coefficients at 238 nm for Co(III) and 332 nm for Ni(II), respectively. A plot of [DNA]/(*ɛ*_b_ − *ɛ*_f_) versus [DNA] gave a slope of 1/(*ɛ*_b_ − *ɛ*_f_) and a *y* intercept equal to 1/*K*_b_(*ɛ*_b_ − *ɛ*_f_), where *K*_b_ is the ratio of the slope to the *y* intercept.

Viscosity measurements were carried out using a semimicro dilution capillary viscometer at room temperature. Each experiment was performed three times and an average flow time was calculated. Data were presented as (*η/η_o_*) versus binding ratio, where *η* is the viscosity of DNA in the presence of complex and *η_o_* is the viscosity of DNA alone.

Thermal denaturation experiments were carried out by monitoring the absorption of CT DNA (50 *μ*M) at 260 nm at various temperatures in the presence (5–10 *μ*M) and the absence of each complex. The melting temperature (*T*_m_, the temperature at which 50% of double-stranded DNA becomes single-stranded) and the curve width (*σ_T_*, the temperature range between which 10% and 90% of the absorption increases occurred) were calculated as reported [23, 24].

The extent of cleavage of super coiled (SC) pUC19 DNA (0.5 *μ*L, 0.5 *μ*g) to its nicked circular (NC) form was determined by agarose gel electrophoresis in Tris-HCl buffer (50 mM, pH 7.2) containing NaCl (50 mM). In the cleavage reactions, the 30 *μ*M and 20 *μ*M complexes in 18 *μ*L buffer were photoirradiated using monochromatic UV or visible light. The samples were then incubated for 1 hour at 37°C followed by addition to the loading buffer containing 25% bromophenolblue, 0.25% xylene cyanol, 30% glycerol (3 *μ*L), and finally loaded on 0.8% agarose gel containing 1.0 *μ*g/mL ethidium bromide. Electrophoresis was carried out at 50 V for 2 hours in Tris-borate EDTA (TBE) buffer. Bands were visualized by UV light and photographed to determine the extent of DNA cleavage from the intensities of the bands using UVItec Gel Documentation System. Due corrections were made for the trace of NC DNA present in the SC DNA sample and for the low affinity of EB binding to SC DNA in comparison to the NC form. The wavelength used for the photo-induced DNA cleavage experiments were 365 nm.

## 3. RESULTS AND DISCUSSION

### 3.1. Characterization of complexes

The elemental analysis data, IR, ^1^H NMR, and magnetic moment data of the new complexes are summarized in Section 2. These data agreed with the theoretical values within the limit of experimental error. These new complexes are insoluble in water, but they are soluble in DMF, DMSO, and in buffer (pH 7.2) solution. The conductometric measurement values in DMF indicate their nonelectrolytic nature.

The IR spectra of ligand qbdp show a strong band in the range 3450 cm^−1^ assigned to *γ*(NH) group. In Co(III) and Ni(II) complexes, this band is absent due to by-coordination of nitrogen atom to the metal ion. Besides, the complexes show new bands at 420 cm^−1^ for (M−N) bond. In addition, the IR spectrum of the PF_6_ salt of each complex showed a strong band in the region 837–839 cm^−1^ ascribable to the counter anion and this band was absent for the corresponding chloride salts [25]. In the ^1^H NMR spectra of the Co(III) complex, the peaks due to various protons of bpy and qbdp ligands are seen to be shifted in complexation with corresponding free ligands, suggesting complexation. Unlike the cobalt(III) complexes, which are diamagnetic, nickel(II) complex was found to be paramagnetic with *μ*_eff_ value of 2.77 ± 0.02 B.M. as expected for typical d^8^ system.

### 3.2. DNA binding experiments

#### 3.2.1. Absorption spectral studies

**Figure 3:**
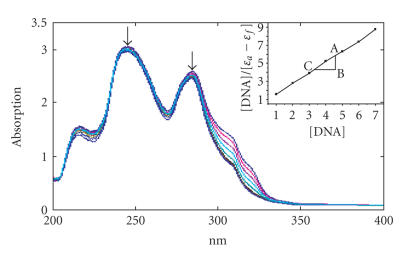
Absorption spectra of complex **(1)** in Tris-HCl buffer upon addition of DNA. [Co] = 0.5 *μ*M, [DNA] = 0–100 *μ*M. Arrow shows the absorbance changing upon the increase of DNA concentration. The inner plot of [DNA]/(*ɛ*_a_ − *ɛ*_f_) versus [DNA] for the titration of DNA with Co(III) complex.

**Figure 4:**
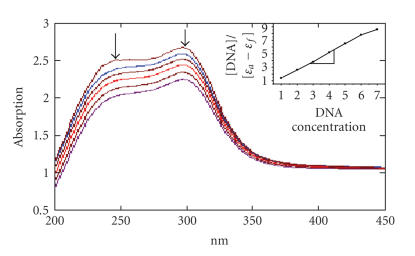
Absorption spectra of complex **(2)** in Tris-HCl buffer upon addition of DNA. [Ni] = 0.5 *μ*M, [DNA] = 0–100 *μ*M. Arrow shows the absorbance changing upon the increase of DNA concentration. The inner plot of [DNA]/(*ɛ*_a_ − *ɛ*_f_) versus [DNA] for the titration of DNA with Ni(II) complex.

In the presence of increasing amounts of CT DNA, both complexes showed hypochromicity and red-shifted charge transfer peak maxima in the absorption spectra. For complexes **(1)** and **(2)**, the hypochromicity observed in the presence of DNA were 22 and 13%, and the bathochromic shifts were 2 and 1 nm (Figures 3 and 4), respectively. The binding constants calculated using (1) are summarized in Table 1. The observed *K*_b_ values for Co(III) and Ni(II) complexes are equal to classical intercalators [EthBr, *K*_b_, 1.8 × 10^6^ M^−1^ in 25 mM Tris-HCl/40 mM NaCl buffer, pH 7.9) and partial intercalating metal complexes [Ru(phen)_2_(dppz)]^2+^, dppz = dipyrido-[3,2-d: 2′,3′-f]-phenazine, *K*_b_ > 10^6^ M^−1^] bound to CT-DNA. So it is obvious that the present complexes are involved in interacalative interactions with CT-DNA. These strongest binding affinities exhibited by these complexes are expected on the basis of the additional aromatic nature of new qbdp ligand.

#### 3.2.2. Viscosity measurements

**Figure 5:**
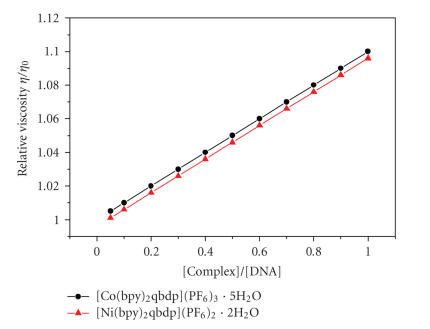
Plot of relative viscosity versus [complex]/[DNA] effect of [Co(bpy)_2_(qbdp)]^3+^ and [NI(bpy)_2_(qbdp)]^2+^ on the viscosity of CT-DNA at 25 (±0.1)°C. [Co(bpy)_2_(qbdp)]^3+^ = 0–100 *μ*M, [NI(bpy)_2_(qbdp)]^2+^ = 0–100 *μ*M, [DNA] = 50 *μ*M.

Furthermore, the interactions between the complex and DNA were investigated by viscosity measurements. Optical photophysical probes provided necessary, but not sufficient, clues to support a binding model. Hydrodynamic measurements that were sensitive to leangth change (i.e., viscosity and sedimentation) were regarded as the least ambiguous and the most critical tests of binding mode in solution in the absence of crystallographic structural data [26, 27]. A classical intercalation model usually resulted in lengthening the DNA helix, as base pairs were separated to accommodate the binding ligand leading to the increase of DNA viscosity. As seen in Figure 5, the viscosity of DNA increased as increasing the ratio of both Co(III) and Ni(II) complexes to DNA. This result further suggested an intercalative binding mode of the complex with DNA and also paralled to the above spectroscopic results, such as hyposhromism and bathochromism of complexes in the presence of DNA.

#### 3.2.3. Thermal denaturation studies

**Figure 6:**
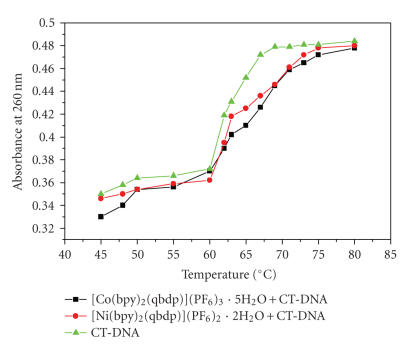
Melting curves of CT-DNA in the absence and presence of complexes.

The DNA thermal melting is a measure of the stability of the DNA double helix with temperature; an increase in the thermal melting temperature (*T*_m_) indicates an interaction between DNA and the metal complex. In the present case, thermal melting studies were carried out at DNA to complex concentration ratios of 25 and *T*_m_ and *σ_T_* (the temperature range between which 10% and 90% of the absorption increase occurred) values were determined by monitoring the absorbance of DNA at 260 nm as a function of temperature. As shown in Figure 6, the *T*_m_ of DNA in the absence of any added drug was found to be 60 ± 1°C, under our experimental conditions. Under the same set of conditions, the presence of complexes **(1)** and **(2)** increased the *T*_m_ by 4 and 2°C, respectively, and the values are given in Table 1.

#### 3.2.4. Cleavage studies by chemical oxidation

**Figure 7:**
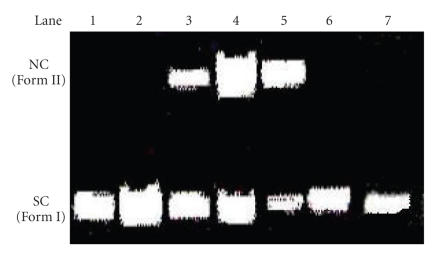
Cleavage of supercoiled pUC19 DNA (0.5 *μ*g) by the cobalt(III) and nickel(II) complexes in a buffer containing 50 mM Tris-HCl and 50 mM NaCl at 37°C. Lane 1: DNA alone; lane 2: DNA + 20 *μ*M of complex (1); lane 3: DNA + 30 *μ*M of complex (1); lane 4: DNA + 40 *μ*M of complex (1); lane 5: DNA + 20 *μ*M of complex (2); lane 6: DNA + 30 *μ*M of complex (2); lane 7: DNA + 40 *μ*M of complex (2). Forms I and II are supercoiled, nicked circular DNA, respectively.

The oxidative DNA cleavage activity of the complexes was studied by gel electrophoresis using super coiled (SC) pUC19 DNA (0.5 *μ*g) in Tris-HCl buffer (pH, 7.2). Both complexes exhibited nuclease activity (Figure 7, lanes 3-4). Control experiment using DNA alone does not show any apparent cleavage (lane 1). At the concentration for 30 *μ*M and 40 *μ*M, the complex **(1)** is able to convert 36% and 71% of the initial SC (Form I) to NC (nicked circular) (Form II), respectively (lanes 3 and 4). Whereas, the complex **(2)** is able to convert 66% of the initial SC (Form I) to NC (Form II) at the concentration of 20 *μ*M (lane 5). From these results, we infer that the complex **(1)** shows more cleavage activity than the complex **(2)**. However, the nature of reactive intermediates involved in the DNA cleavage by the complexes has not been clear yet.

### 3.3. DNA photocleavage

**Figure 8:**
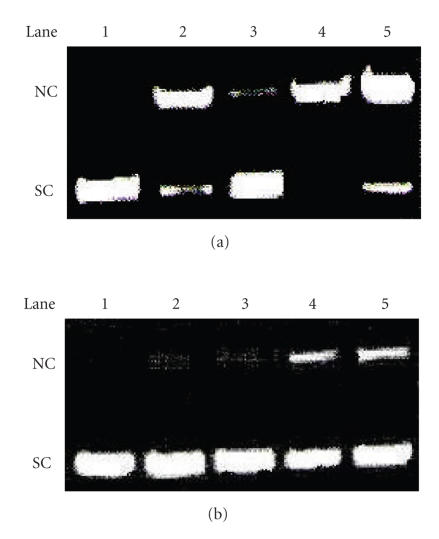
(a) Gel electrophoresis diagram of the control experiments using SC DNA (0.5 *μ*g), **(1)** (30 *μ*M), and other additives at 365 nm for an exposure time of 1 hour. Lane 1: DNA control; lane 2: DNA + DMSO (4 *μ*L) + **(1)**; lane 3: DNA + NaN_3_ (38 *μ*M) + **(1)**; lane 4: DNA + D_2_O (14 *μ*L) + **(1)**; lane 5: DNA + **(1)**. (b) Gel electrophoresis diagram of the control experiments using SC DNA (0.5 *μ*g), **(2)** (30 *μ*M), and other additives at 365 nm for an exposure time of 1 hour. Lane 1: DNA control; lane 2: DNA + DMSO (4 *μ*L) + **(2)**; lane 3: DNA + NaN_3_ (38 *μ*M)+ **(2)**; lane 4: DNA + D_2_O (14 *μ*L) + **(2)**; lane 5: DNA + **(2)**.

The photo-induced DNA cleavage activity of the complexes was studied by gel electrophoresis using supercoiled (SC) pUC19 DNA (0.5 *μ*g) in Tris-HCl buffer (pH, 7.2). Selected DNA cleavage data are given in Table 2, and the gel diagrams are shown in Figure 8. The complex **(1)** (30 *μ*M in 18 *μ*L volume) shows 66% cleavage of the SC DNA, whereas complex **(2)** (20 *μ*M in 18 *μ*L volume) shows 37% of cleavage on 1 hour exposure at 365 nm. Control experiments using qbdp ligand alone do not show any significant cleavage of SC DNA even on long exposure time. The results indicate the important role of metal in these photo-induced DNA cleavage reactions. The complexes show the presence of charge-transfer band near 400 nm. It is likely that the photocleavage at 365 nm involves photoexcitation of the charge-transfer band leading to the formation of an excited singlet state that through the triplet state activates molecular oxygen to form reactive singlet oxygen species. To test the possibility that photoinduced cleavage involves the formation of singlet oxygen, which is known to react with guanine residues at nuetral pH, the cleavage was tested in the presence of D_2_O. Singlet oxygen would be expected to induce more strand scission in D_2_O than in H_2_O due to its longer lifetime in the former solvent [28–31]. Control experiments show that the singlet oxygen quencher sodium azide significantly inhibits the cleavage reaction, while the hydroxyl radical scavenger DMSO has no apparent effect on the cleavage process. The formation of singlet oxygen is further supported by the enhancement of the percentage of SC DNA cleavage in D_2_O solvent. From the above results, we conclude that in the photocleavage activity of complexes **(1)** and **(2)** at 365 nm, complex **(1)** shows significantly higher cleavage activity than complex **(2)** based on its DNA binding propensity.

## 4. CONCLUSION

The new mixed ligand cobalt(III) and nickel(II) complexes have been synthesized and characterized. The DNA binding properties of these two complexes were studied by using absorption spectra, viscosity, and thermal denaturation experiments. The results show that the complexes were interacting with the CT-DNA. We also carried out the DNA cleavage by oxidative as well as photo-irradiations. The cleavage study results show that the Co(III) complex is more nuclease than the Ni(II) complex.

## Figures and Tables

**Table 1:** DNA binding constant and melting-temperature data.

Complex	*K*_b_ (M^−1^)	*T*_m_ (°C)	*σ*_T_ (°)

[Co(bpy)_2_qbdp](PF_6_)_3_ · 5H_2_O	1.3 × 10^6^	64	25
[Ni(bpy)_2_qbdp](PF_6_)_2_ · 2H_2_O	3.1 × 10^5^	62	21

**Table 2:** Selected DNA (SC pUC19 DNA, 0.5 *μ*g) cleavage data of complexes **(1)** and **(2)** in Tris-buffer (pH 7.2), Form I and Form II are SC and NC forms of DNA, respectively.

Reaction condition	*λ* (nm)	*t* (h)	[Complex] (*μ*M)	Form I (%)	Form II (%)

DNA control	365	1.0	—	93	7
DNA + **(1)**	365	1.0	30	34	66
DNA control	365	1.0	—	98	2
DNA + **(2)**	365	1.0	20	63	37
DNA + D_2_O (14 *μ*L) + **(1)**	365	1.0	30	10	90
DNA + D_2_O (14 *μ*L) + **(2)**	365	1.0	20	89	11
DNA + DMSO (4 *μ*L) + **(1)**	365	1.0	30	29	71
DNA + DMSO (4 *μ*L) + **(2)**	365	1.0	20	72	28
DNA + NaN_3_ (38 *μ*M) + **(1)**	365	1.0	30	89	11
DNA + NaN_3_ (38 *μ*M) + **(2)**	365	1.0	20	90	10

*λ*: excitation wavelength.

*T*: exposure time.
